# Treating a Non-mixed Neuroendocrine Colorectal Tumor Under Mixed Neuroendocrine Neoplasm (MiNEN) Protocols: A Case Report of Successful Outlier Management

**DOI:** 10.7759/cureus.79389

**Published:** 2025-02-20

**Authors:** Taimur Aslam, Akash Sankar Uday Vankayala, Sabeen Jafri, Yashna Singh, Muhammad Niazi, Yevgeniy Skaradinskiy

**Affiliations:** 1 Internal Medicine, Staten Island University Hospital, New York, USA; 2 Internal Medicine, Touro College of Osteopathic Medicine, New York, USA; 3 Pathology and Laboratory Medicine, Staten Island University Hospital, New York, USA; 4 Oncology, Staten Island University Hospital, New York, USA

**Keywords:** adenocarcinoma, colorectal cancer, lymph node metastasis, mixed adenocarcinoma-large cell neuroendocrine carcinoma, neuroendocrine carcinoma, rectal mass

## Abstract

A 65-year-old male presented after a positive Cologuard testing. He was asymptomatic at presentation, with no reported complaints of fever, chills, abdominal pain, diarrhea, constipation, hematochezia, or unexpected weight loss. CT imaging of the abdomen and pelvis revealed a 2 cm mass at the rectosigmoid junction with a single enlarged lymph node nearby measuring 2.7 x 1.6 cm, raising suspicion for regional metastatic adenopathy. He underwent a colonoscopy, which confirmed a rectosigmoid mass. Biopsy results showed adenocarcinoma of the colon. Consequently, the patient was scheduled for a laparoscopic sigmoidectomy with low anterior resection. The pathology of the surgical specimen confirmed adenocarcinoma, moderately differentiated, associated with a component of poorly differentiated large cell neuroendocrine carcinoma, with the neuroendocrine component best developed in the lymph node metastasis. Although the patient did not meet the standard criteria for mixed neuroendocrine-non-neuroendocrine neoplasm (MiNEN) tumors, treatment was administered using chemotherapeutic agents typically reserved for MiNENs. He was treated with platinum-based doublet with good response and has been in remission for one and a half years. This report highlights the importance of flexible therapeutic approaches when pathology marginally deviates from the standard established criteria, illustrating that tailoring treatment to the specific pathology can provide considerable patient benefits.

## Introduction

Neuroendocrine tumors (NETs) are rare neoplasms that can arise throughout the body, with variable incidence depending on the site. Gastrointestinal (GI) NETs are neoplasms derived from the enterochromaffin-like cells (ECL cells) of the bowel mucosa showing morphologic and immunophenotypic signs of neuroendocrine differentiation. They occur throughout the GI tract in the stomach, small bowel, and colon, most commonly in the small intestine and rectum [1,2]. The presentation of colorectal neuroendocrine neoplasms (NENs) may be asymptomatic or include very nonspecific symptoms such as pain, perianal bulge, anemia, and bloody stools [3]. These tumors are characterized immunohistochemically by positivity for synaptophysin, chromogranin A, B, C, neuron-specific enolase, and CD56 [4]. Their diagnosis can be challenging due to overlapping features with adenocarcinomas, requiring a high degree of suspicion and appropriate immunohistochemical staining.

The incidence of colorectal NETs has increased over the past years, especially in rectal tumors, with the advancements in endoscopy and technical refinements [2]. NETs are aggressive and associated with earlier metastases and poor outcomes compared to adenocarcinomas [5]. Hence, the management requires a multidisciplinary approach with aggressive surgery and chemotherapy, often utilizing regimens beyond traditional 5-fluorouracil (5-FU)-based therapy [6]. We discuss a case that highlights the atypical presentations of rare colon cancer subtypes and describe a therapeutic approach that varies from the one used for typical colorectal adenocarcinomas. An awareness of the condition's varied presentations and a prompt diagnosis are critical given the poor prognosis. We report a case of a 65-year-old male diagnosed with mixed adenocarcinoma and neuroendocrine tumor that was treated on the lines of mixed neuroendocrine-non-neuroendocrine neoplasm (MiNEN) with very good outcomes. We also highlight the challenges in the diagnosis and management of these cases in community settings.

## Case presentation

The patient was a 65-year-old male who presented after a positive Cologuard testing. He was asymptomatic at presentation, with no reported complaints of fever, chills, abdominal pain, diarrhea, constipation, hematochezia, melena, or unexpected weight loss. Initial CT imaging of the abdomen and pelvis revealed a 2 cm mass in the upper rectum and junction of the sigmoid colon with a single enlarged lymph node nearby measuring 2.7 x 1.6 cm, which was suspicious for regional metastatic adenopathy. He underwent a colonoscopy, which confirmed a large, irregular, tan, ulcerated rectosigmoid mass 18 cm from the anal verge. A colonoscopic biopsy demonstrated adenocarcinoma of the colon. The patient was scheduled for a laparoscopic sigmoidectomy with low anterior resection, and pathology revealed the size of the tumor as 3.2 x 2.4 x 1.4 cm, invading through muscularis propria into pericolic tissue with presence of cancer in single regional lymph node, out of total 13 lymph nodes removed: stage III T3N1M0 as per tumor node metastasis (TNM) staging for colorectal cancer, American Joint Commission on Cancer/Union for International Cancer Control (AJCC /UICC) 8th edition.

**Figure 1:**
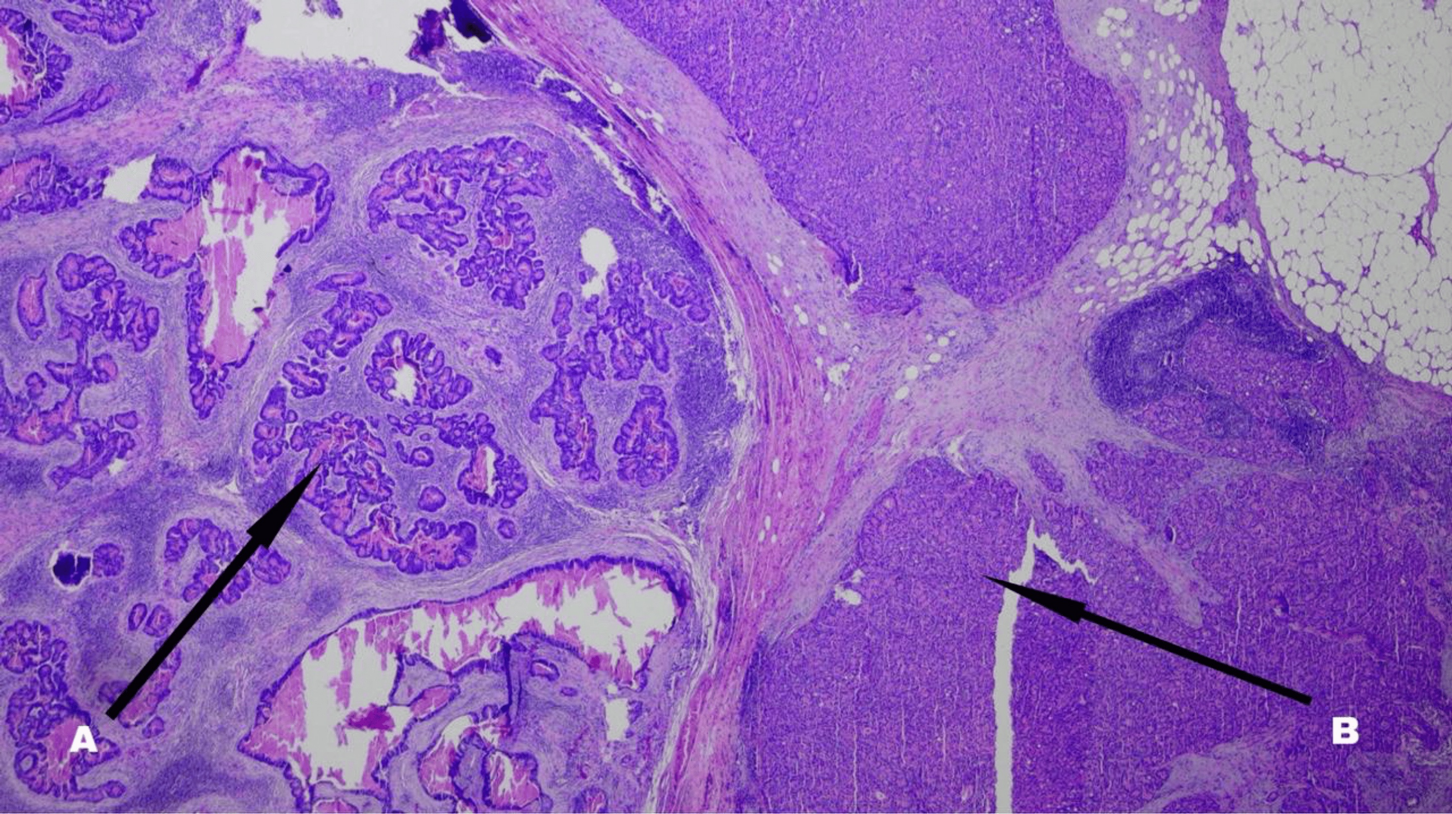
Adenocarcinoma, moderately differentiated, associated with a component of poorly differentiated large cell neuroendocrine carcinoma Note the adenocarcinoma component on the left side (A) and the neuroendocrine carcinoma component on the right side (B)

**Figure 2:**
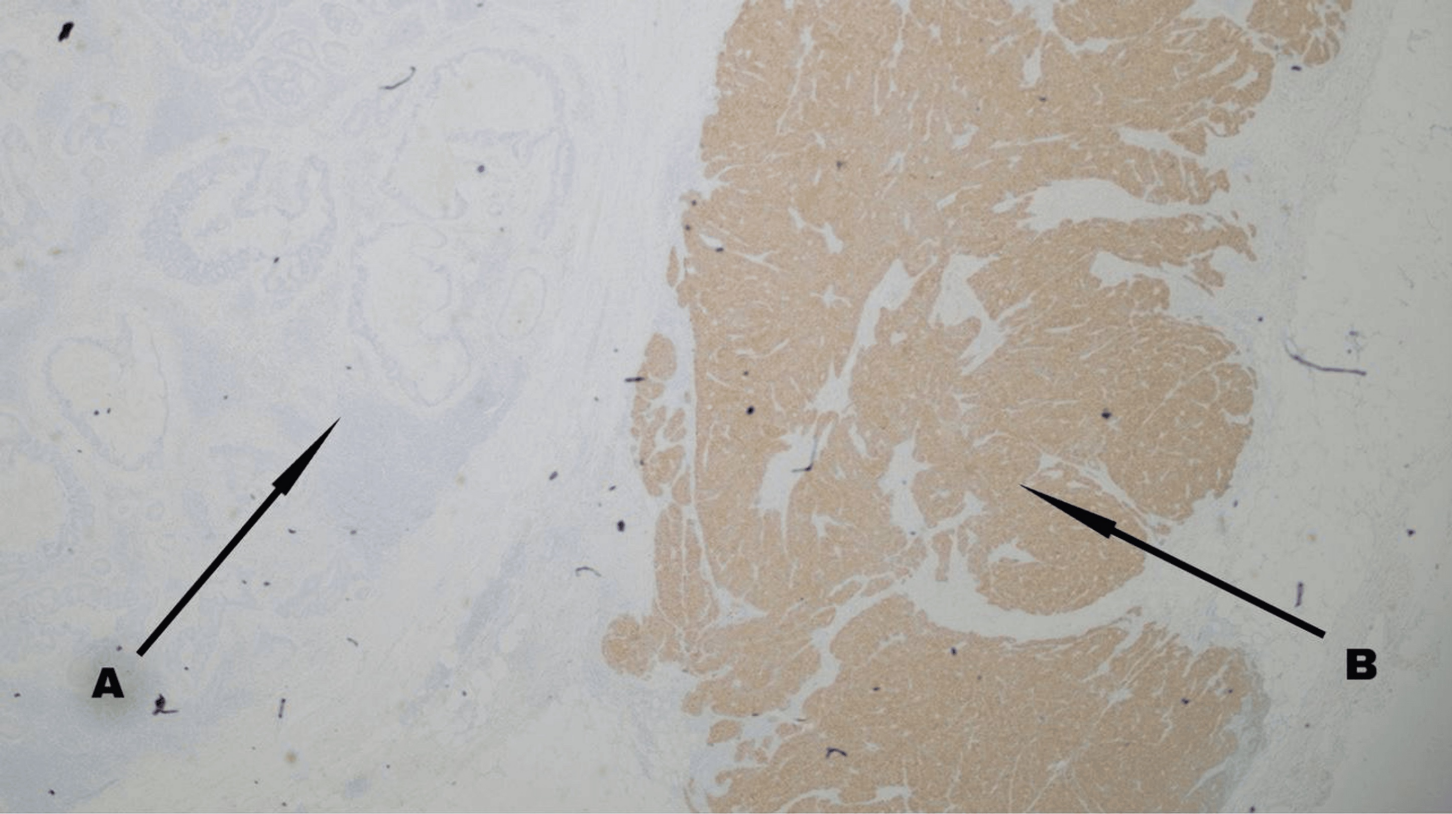
Immunoreactivity for synaptophysin Diffuse and regular staining appears in the cytoplasm of neuroendocrine cells. The adenocarcinoma component (A) remains unstained, contrasting with the neuroendocrine carcinoma component (B)

**Figure 3:**
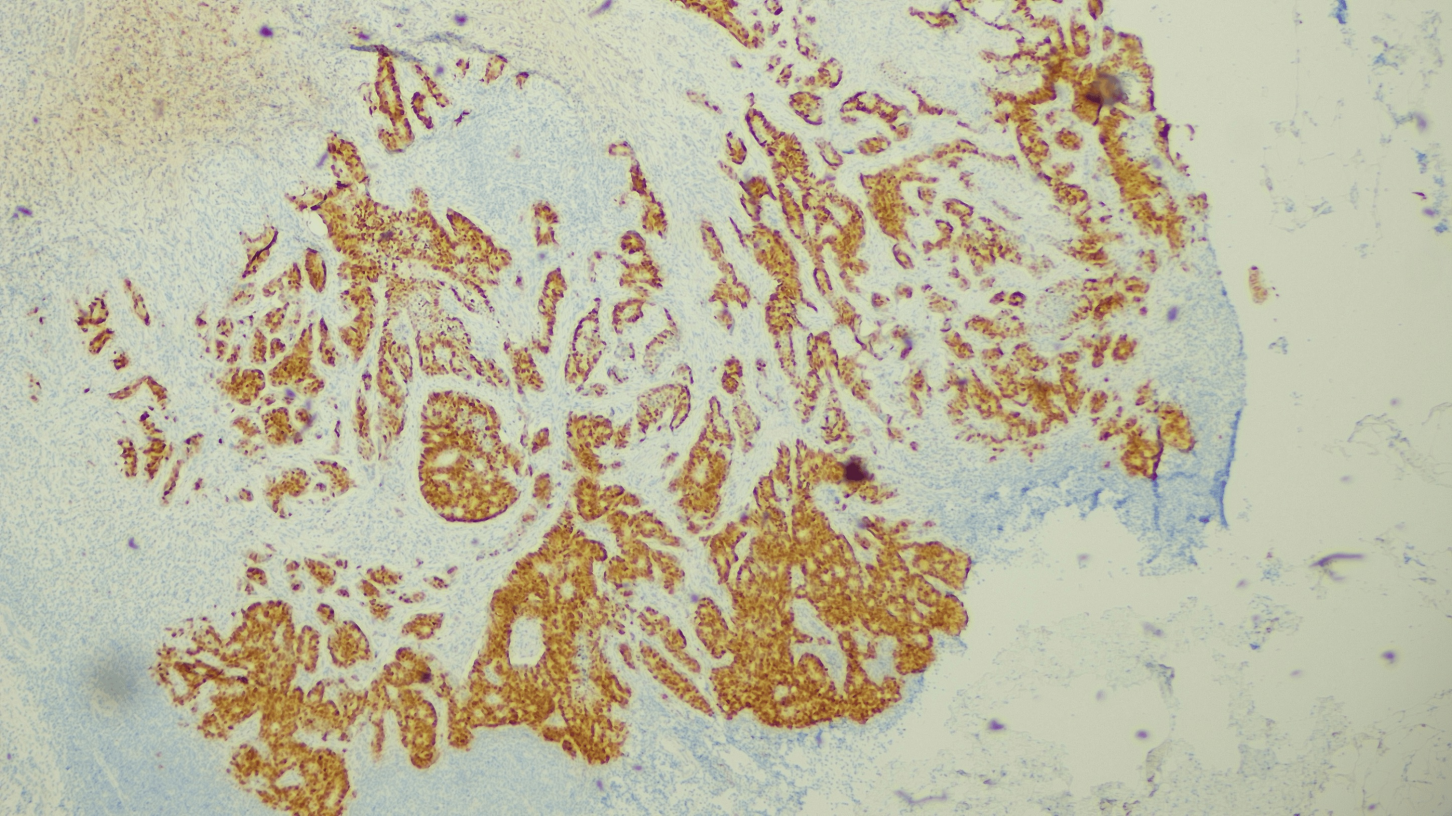
Immunoreactivity for CDX2 The adenocarcinoma shows positive staining for CDX2

The pathology seen in the sections (Figures 1-3) from the primary site was consistent with a conventional type of moderately differentiated adenocarcinoma, with only a focal suggestion of possible neuroendocrine differentiation (with subtle morphological changes and some chromogranin staining). In the positive lymph node, there was a distinct component with morphological and immunohistochemical characteristics most indicative of large cell neuroendocrine carcinoma (tumor being positive for both chromogranin and synaptophysin). The overall appearance suggested that the neuroendocrine carcinoma component was likely histogenetically related to the adenocarcinoma (i.e., likely a mixed tumor). The presence of a distinct neuroendocrine carcinoma component in the lymph node carried the same implication as a typical MiNEN whereby the two malignant components are both present within the primary tumor mass. Accordingly, this tumor was regarded as a MiNEN when contemplating strategies for adjuvant therapy.

The patient was initiated on a chemotherapeutic regimen of carboplatin and etoposide and completed six cycles in four months. Carboplatin was used instead of cisplatin because of compromised renal function. On follow-up CT/positron emission tomography (CT/PET) scans, and colonoscopy, no evidence of residual or metastatic disease was noted. The patient has been in remission for the past 18 months now.

## Discussion

NETs are exceedingly rare and account for less than 1% of all colorectal tumors. They are neoplasms derived from the ECL cells of the bowel mucosa showing morphologic and immunophenotypic signs of neuroendocrine differentiation They occur throughout the GI tract in the small bowel, stomach, and colon, most commonly in the cecum and rectum [1,2]. The presentation of colorectal NENs may be asymptomatic or very nonspecific symptoms such as pain, perianal bulge, anemia, and bloody stools. The molecular pathogenesis of these tumors is increasingly yet incompletely understood [7]. Colonic NETs exhibit distinct biological differences from traditional adenocarcinomas that impact treatment approaches. Unlike adenocarcinomas, colonic NETs overexpress DNA repair enzymes, conferring relative platinum resistance. Additionally, molecular alterations in p53 and retinoblastoma (RB) reduce sensitivity to 5-FU-based chemotherapy [8].

These tumors tend to be rapidly progressive with high malignancy potential compared to colorectal adenocarcinomas [9], and an aggressive multidisciplinary treatment approach is typically required. The prognosis for GI NETs depends on the tumor location with a five-year disease-free survival of 95% for the rectal variant, 90% for the appendix, 86% for the small intestine, 82% for the stomach, and 67% for the colonic [10]. The median survival varies significantly by tumor location, being favorable for rectal NETs (>20 years), intermediate for cecal NETs (~9 years), and poor for colonic NETs (~1 year) [2]. Ki-67 can have an important prognostic role when evaluated in the NET component of the tumor. Studies have shown that patients with Ki-67 <55% were associated with a favorable prognosis [11]. Moreover, cases with NET components that exhibit microsatellite instability (MSI) are associated with favorable outcomes with immunotherapy. This makes it customary to send these tumors for MSI testing [12]. Other factors associated with worse prognosis in these tumors include lymph-vascular invasion, tumor size, and level of tumor infiltration [13].

The standard adjuvant regimen for resected colon adenocarcinoma is 5-FU and leucovorin based on robust data demonstrating improved survival compared to surgery alone. However, studies have shown poorer responses to 5-FU-based therapy as mentioned earlier [1]. In contrast, platinum agents like cisplatin and carboplatin, which are not part of standard management for colon adenocarcinoma, have shown a greater efficacy in NETs. Compared to 5-FU, platinum appears to have superior activity in colonic NETs, likely related to fewer DNA repair defects in this cancer versus adenocarcinoma. While chemotherapy, particularly platinum-based regimens, has been shown to increase survival in advanced-stage, high-grade NETs, the efficacy remains variable regarding other combination regimens with etoposide. For instance, a randomized clinical trial comparing etoposide plus cisplatin (EP) and irinotecan plus cisplatin (IP) in advanced NETs of the digestive system showed a median survival of 12.5 months in the EP arm and 10.9 months in the IP arm (HR: 1.04; 90% CI: 0.79-1.37) [14]. Another ongoing SONNET phase II trial is investigating the disease control rate of lanreotide + temozolomide (TEM), highlighting the need for further research into systemic therapies [3].

Nevertheless, when possible, surgical removal of the tumor is often considered to be the main treatment for NETs of the colon. Endoscopic mucosal resection (EMR) and underwater EMR (uEMR) are good enough treatment modalities for localized NENs. However, T2 or N+ stage lesions should be accurately studied by total body imaging such as 68-Ga-DOTATATE-PET and CT scanning to determine metastasis and may require lymph node dissection [1]. Further biological profiling and clinical trials are needed to optimize systemic therapy for this aggressive disease. FDA has approved cancer antigen 19-9 (CA 19-9) as a biomarker for routine monitoring of pancreatic cancer. Of note, elevated CA 19-9 can have significant prognostic values in cases where a preoperative carcinoembryonic agent (CEA) is not elevated [15]. As for second-line treatment options, there is no standard therapy available because of the scarcity of this condition. However, FOLFOX or FOLFIRI +/- bevacizumab, capecitabine/Temodar can be considered reasonable second-line options [16]. Next-generation sequencing (NGS) should be sent for in all patients with metastatic disease, to employ targeted therapy options from the armamentarium [17].

In the present case, the rectal tumor had adenocarcinoma and neuroendocrine components but did not fulfill the WHO criteria for MiNEN; yet, the patient was successfully treated as a case of MiNEN with platinum-based chemotherapeutic agents. The case was challenging as to which chemotreatment modalities should be tried, i.e., 5-FU vs. platinum-based agents. The successful response of this patient's tumor to MiNEN-based chemotherapy agents, despite not meeting the WHO criteria for MiNEN, opens up a new avenue for the discussion of the management of similar cases in the future. The presence of a focal NET component within both the colonic mass and a metastatic lymph node, histologically linked to the adenocarcinoma, poses a clinical challenge. This discovery underscores the intricate nature of these cancers, which can exhibit aggressive behavior, distinct from classic colorectal adenocarcinomas, necessitating tailored management strategies. 

## Conclusions

This report highlights the atypical presentation of a rare and aggressive subtype of colorectal cancer which had both components of colorectal adenocarcinoma and neuroendocrine carcinoma components but did not meet the WHO criteria for MiNENs. Despite this, the decision to treat this patient as a case of MiNEN with platinum-based chemotherapy agents led to the successful remission of the tumor. This report emphasizes that the aggressive nature of NETs should be considered when adopting treatment strategies for tumors with pathological findings that deviate from the standard established criteria. Embracing adaptability and flexibility in the management of these complex tumors can have significant therapeutic relevance and lead to overall better outcomes. This report underscores the importance of clinician awareness, prompt diagnosis, and the need for ongoing clinical trials to better understand and manage mixed NETs of the colon.
